# Non-neutralizing functions in anti-SARS-CoV-2 IgG antibodies

**DOI:** 10.1016/j.bj.2023.100666

**Published:** 2023-09-29

**Authors:** Sebastian Reinig, Shin-Ru Shih

**Affiliations:** a Research Center for Emerging Viral Infections, College of Medicine, Chang Gung University, Taoyuan, Taiwan; b Department of Laboratory Medicine, Linkou Chang Gung Memorial Hospital, Taoyuan, Taiwan; c Department of Medical Biotechnology and Laboratory Science, College of Medicine, Chang Gung University, Taoyuan, Taiwan; d Research Center for Chinese Herbal Medicine, Chang Gung University of Science and Technology, Taoyuan, Taiwan

**Keywords:** IgG, COVID-19, Vaccines, Glycosylation, Isotype, Fc

## Abstract

Most individuals infected with or vaccinated against COVID-19 develop antigenic neutralizing immunoglobulin G (IgG) antibodies against the SARS-CoV-2 spike protein. Although neutralizing antibodies are biomarkers of the adaptive immune response, their mere presence is insufficient to explain the protection afforded against the disease or its pathology. IgG exhibits other secondary effector functions that activate innate immune components, including complement, natural killer cells, and macrophages. The affinity for effector cells depends on the isotypes and glycosylation of IgG antibodies. The anti-spike IgG titer should be sufficient to provide significant Fc-mediated effects in severe COVID-19, mRNA, and protein subunit vaccinations. In combination with aberrant effector cells, pro-inflammatory afucosylated IgG1 and IgG3 may be detrimental in severe COVID-19. The antibody response of mRNA vaccines leads to higher fucosylation and a less inflammatory IgG profile, with a long-term shift to IgG4, which is correlated with protection from disease.

## Introduction

SARS-CoV-2 is a coronavirus and the causative agent of COVID-19 disease. In most infected individuals, COVID-19 leads to self-limiting symptoms; however, in a minority of patients, it can lead to hospitalization and even death [1]⁠. Severe COVID-19 is characterized by hyperinflammation, cytokine storm, hypercoagulability, tissue damage, and fibrosis in later stages [2]⁠. As prevention for the development of severe disease or infection, most countries employed mass vaccination [3]⁠. Most individuals infected with SARS-CoV-2 or vaccinated against COVID-19 develop antigen-specific antibodies (immunoglobulins), against the virus as part of their adaptive immune system [4,5]⁠. There are different immunoglobulin classes, including IgM, IgG, IgA, and IgE. Immunoglobulin G (IgG) represents the main anti-SARS-CoV-2 antibody [6]⁠. Many studies have focused on neutralizing antibodies, which have the capability to block the entry of the virus into the target cells. In vaccinated individuals, the neutralizing antibody correlates well with protection [7]⁠. However, there are also conflicting data, indicating either no significant neutralizing antibodies or even detrimental effects. Patients with X-linked agammaglobulinemia, who are unable to produce antibodies, are not more susceptible to diseases caused by enveloped viruses like SARS-CoV-2 [8,9]⁠. In immune-naive individuals, higher levels of anti-SARS-CoV-2 IgG are associated with more severe disease [4]⁠. Since other parts of the immune system, especially T-cell-mediated immunity, play a significant role in immunity against coronaviruses [10]⁠, the individual contribution of neutralizing antibodies is difficult to determine. Furthermore, beyond the neutralizing function, IgG can also exhibit secondary effector functions by activating parts of the innate immune system and can lead to either the phagocytosis of viral particles or the elimination of infected cells [11]. In coronaviruses and other respiratory viruses, antibodies can also enhance the disease, either by facilitating the entry of the virus or by inducing hyper-inflammation [12,13]⁠. This review will dissect the evidence to determine the conditions under which the IgG-mediated response and their Fc-mediated functions against SARS-CoV-2 have a protective, detrimental, or insignificant impact.

## Fc and Fc-receptor interaction

**Fig. 1:**
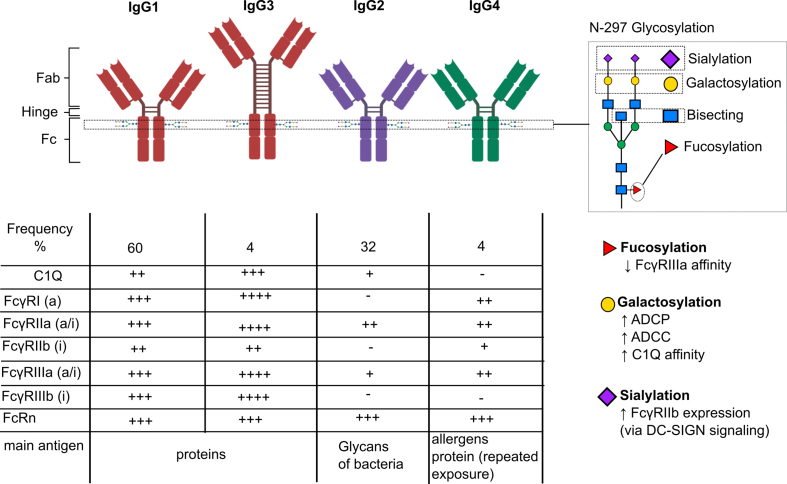
IgG subclass and N-297 glycosylation in the Fc-domain determine the affinity of IgG to the C1Q of the complement system and the Fc receptors. This will finally determine the activity of antibody-mediated responses, including antibody-dependent cellular phagocytosis (ADCP), antibody-dependent cellular cytotoxicity (ADCC), complement activation, and inhibitory functions. (+) affinity to Fc receptor and C1Q, adapted from Ref. [15]⁠ (A): cell activation, (i): cell inhibition.

IgG antibodies bind to their specific antigens by one or both Fab domains. The crystallizable fragment (Fc) domain can bind to different Fc receptors [Fig. 1]. Fc receptors can be activating or inhibitory. This is dependent on the Fc receptor, as well as downstream pathways. Fc-signaling has been extensively reviewed [14]⁠. The IgG is subdivided into different subclasses, which differ in their affinity for the Fc receptor [15]⁠. In viral infections, IgG1 and IgG3 are the dominant IgG subclasses, which usually bind to viral surface proteins and have the strongest affinity for Fc receptors. IgG2 is the only IgG subclass whose production is T-cell-independent, and its antigens are usually bacterial polysaccharides. IgG4 is usually expressed after continuous exposure to antigens, and it has the lowest affinity for Fc receptors and no affinity for the complement system; therefore, it conveys immune tolerance. Viral infections usually only appear in latent and chronic infections. Another important structural feature determining the affinity of the Fc receptor is conserved n-297 glycosylation [16]⁠. Most research on IgG glycosylation has focused on the IgG1 subclass. Antibody glycosylation is processed in the Golgi apparatus, and switching of the IgG subclass can occur through the sequential recombination of heavy-chain genes in B-cells. Activation and class switching require antigen-induced activation via B-cell receptors and co-factors, including toll-like receptor (TLR) stimulation, T-cell binding, and cytokine signaling. However, the signal transduction leading to a specific class switch is poorly understood [17]⁠. The regulation of IgG glycosylation is not well understood either. The few studies that have been conducted in mice in this regard have shown that adjuvants and cytokines influence IgG glycosylation [18,19]⁠.

## Antibody mediated secondary effector functions

**Fig. 2:**
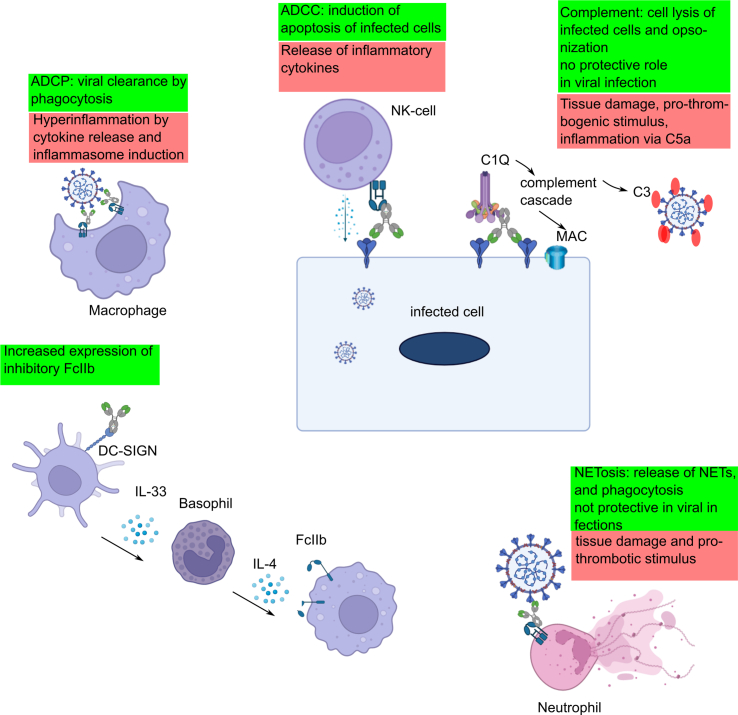
Fc-mediated functions and their effector cells for ADCP by macrophages. The anti-inflammatory and protective functions are listed in green. The potential negative effects are described in red.

Various antibody-mediated functions are elicited by binding to their effectors [Fig. 2]. Various in vitro assays have been established [Table 1] to measure the immune response of immune cell lines or leukocytes to immunocomplexes formed by antigen-specific antibodies and either the virus, antigen-expressing cells, or the antigen alone. In vivo assays are usually restricted to comparing the treatment with mono- or polyclonal antibodies that have wild-type or modified Fc domains, which may have either loss or gain of function.

**Table 1:** In vitro assays were used in the studies cited in this review to measure antibody-mediated functions.

Function	Assay type	Description
Fcγ-affinity	ELISA	Bind antibodies to immobilized antigens and add to the biotinylated Fc receptor. With enzyme-linked streptavidin, the binding can be assayed [82]
	Flow cytometry	Beads or cells with the specific Fc receptor or antigen are incubated with serum or antibodies and then identified by co-immunostaining [87]
Complement	Bead based	Fluorescent beads with the antigen are incubated with antibodies. Complement is added. C3 fluorescent immunostaining is performed and complement coated beads are identified by flow cytometry. [87]
	Cell based	Target cells with a fluorescent viability marker expressing the antigen are incubated with serum or plasma, and the viability is measured by flow cytometry [26]
	ELISA	C1Q binding assay for antibodies [85]
ADCP	Flow cytometry	Coincubation of fluorescent antibody-antigen-coated beads The fraction of cells with fluorescent beads is measured by flow cytometry [60]
ADCC	Effector cell based	Natural killer cells are incubated with antigen–antibody immunocomplexes. CD107a is used as a marker for NK-cell activation in flow cytometry [87].
	FcγRIIIa signaling	A reporter cell line expressing FcγRIIIa and luciferase by FcγRIIIa signaling is incubated with immunocomplexes. The fluorescence is measured as ADCC activity [26]
	Target cell based	Target cells expressing the antigen are incubated with antibodies. Effector cells are co-incubated, and cell death is measured as an indicator for ADCC activation [26, 40]
	ELISA	FcγRIIIa affinity is used as a proxy for NK-cell activity [82]
Antibody mediated inflammation	Cell based	Either immobilized antigen or pseudovirus with antigen are incubated with antibodies and, when coincubated with effector cells, The cytokine release is then measured by ELISA in the supernatant [27, 45].

### Antibody-dependent cellular phagocytosis (ADCP)

The phagocytosis of pathogens and immune complexes with IgG, IgA, or IgM is performed by macrophages, monocytes, dendritic cells, or neutrophils. The immune complex is then further degraded in lysosomes. This can lead to the clearance of the virus and can help stimulate T-cells via antigen presentation. In several viral infections, like HIV or influenza virus, it was shown that the ADCP function is important for viral clearance [20]⁠. In some situations, phagocytosis is also exploited by viruses. With subneutralizing titers, some viruses can also use this phagocytosis process by using Fc binding to infect macrophages, subsequently using them for further spread. This phenomenon is well known for the dengue fever virus and some coronaviruses [12]⁠. SARS-CoV-2 can infect macrophages via Fc receptors and antibody-mediated functions; however, the infection is abortive and cannot replicate in macrophages. However, the infection can still lead to the formation of the inflammasome, alter the macrophages, and may contribute to the hyperinflammatory state [21]⁠. Furthermore, the viral antigens and debris are not degraded in the macrophages in some patients, even more than 15 months after the initial infection, and are associated with post-acute sequelae [22,23]⁠. However, the exact role of ADCP, whether it is protective or, under certain circumstances, harmful, needs to be elucidated.

### Antibody-dependent cellular cytotoxicity (ADCC)

In ADCC, antibodies bind to the antigen on the surface of a cell, forming an immune complex. Via the FcγIIIa receptor, the effector cell binds to the immune complex and releases perforin and granzyme to induce apoptosis in virus-infected cells, but it can also be directed against cancer cells. Natural killer (NK) cells are classical inducers of ADCC. The lack of n-297 fucosylation (afucosylation) increases the affinity for FcγIIIa [24]⁠. To a lesser degree, galactosylation increases the affinity of IgG to FcγIIIa and therefore the potency to induce ADCC [25]⁠. In viral infections of influenza and HIV, ADCC is associated with protection [11]⁠, whereas in COVID-19 disease, the ADCC potency of SARS-CoV-2 IgG is associated with more severe disease [[26], [27], [28]]⁠. The discussion of whether and how this stronger ADCC potency is potentially detrimental is discussed in detail in the section: IgG response in natural infection and COVID-19 disease.

### Complement system

The complement system is a part of the innate immunity system and represents a group of plasma proteins that activate a signaling cascade with C3 as the central effector, leading to various effects, from cell lysis to cytokine release and opsonization. IgM and IgG antibodies can activate the complement cascade by binding to C1Q. This can lead to the lysis of infected cells or enveloped viruses by forming the MAC complex (ADCD) or facilitate phagocytosis via C3 deposition. IgG1 and IgG3 are the main complement cascade-inducing IgG isotypes [29]⁠. Furthermore, IgG4 with non-galactosylated n-297 glycans can activate the complement system via the lectin pathway [30]⁠. Deficiency of the complement system is linked to a higher susceptibility to bacterial infections but not viral infections. On the other hand, complement activation is associated with severe disease in COVID-19, and a mouse model deficient in C3 is protected from the severe disease caused by SARS-CoV-1 [29]⁠. However, it is not clear whether this is caused by the antibody-mediated function or the virus and its antigens themselves, since the virus can also directly activate the alternative or the lectin pathway [29]⁠.

### Neutrophil mediated antibody function

Neutrophils represent 50–70% of all leukocytes. Upon binding of the antibody–immune complex, they engage in phagocytosis, releasing reactive oxygen species (ROS) and neutrophil extracellular traps (NETs) composed of chromatin and antimicrobial proteins. Neutrophil activation and NETosis are associated with severe disease in COVID-19. There are elevated levels of immature neutrophils in COVID-19 patients with NETosis. Furthermore, the neutrophils of COVID-19 patients are more sensitive to NETosis-activating stimuli. Similar to the complement system, SARS-CoV-2 can directly activate neutrophils, and the contribution of antibody-mediated neutrophil activation remains unclear [31]⁠.

## IgG response in natural infection and COVID-19 disease

SARS-CoV-2 infects the host cell via the spike (S) glycoprotein. The S glycoprotein is a trimeric protein consisting of S1 and S2 units. The S1 unit consists of the N-terminal domain and receptor-binding domain (RBD) and is attached to angiotensin-converting enzyme 2 (ACE2) via the RBD. Upon binding, S1 and S2 are cleaved at the furin cleavage site, triggering the fusion of the virus with the cell membrane [32]⁠. Based on a human challenge study of SARS-CoV-2 infection, symptoms appear after 2–4 days [33]⁠. Viremia lasts around 9 days after onset of symptoms [33,34]⁠. If the disease progresses to a more severe stage, like pneumonia, symptoms persists even after the decline of viremia. At this stage, the disease may manifest with severe and potentially fatal complications, including pneumonia, ARDS, hypercoagulation, cytokine storms, and hyperinflammation [2]⁠.

### IgG kinetics in natural infection

Anti-SARS-CoV-2 IgG increases 7–11 days after the onset of symptoms. This is after the decline of viremia and, therefore, should not have a significant contribution to the viral clearance in the immune-naive individual [33,35,36]⁠. During natural infections, the frequency of developing neutralizing antibodies increases with increasing disease severity, ranging from 14% in asymptomatic patients to 89.3% in severe patients [37]⁠. The main targets of anti-SARS-CoV-2 IgG are the S and N proteins. The anti-S antibodies exhibit a longer half-life in the serum in the range of 140–225 days versus 68–71 days for anti-N IgG [38,39]⁠. The neutralization titer wanes here faster than the Fc-mediated functions [40]⁠. Antibodies against other structural proteins like the membrane (M) and enveloped (E) proteins are only found in a fraction of infected individuals [6,41]⁠. There is an increased IgG titer with disease severity shown in multiple studies [4]⁠. The main IgG subclasses are IgG1 and IgG3 in mild and severe disease, respectively, and the IgG3 proportion is higher in severe disease, although it decreases over time [6,27,39,42]⁠. Anti-SARS-CoV-2 IgG2 and IgG4 were detected in a minority of patients (21% and 9%, respectively) [39]⁠.

### IgG effector functions and its relation to severe COVID-19 disease

In general, the potency of all antibody-mediated functions, including ADCC, neutrophil activation, complement, and ADCP, is elevated in severe versus moderate COVID-19 disease [[26], [27], [28],^43^]⁠. This could be a simple reflection of the higher antibody levels in severe patients. However, only NK-cell-related functions are significantly higher after 14 days of symptom onset in severely ill patients [28]⁠. Several studies have consistently shown that IgG from severely ill patients needing hospitalization has a higher potential to induce ADCC [27,28]⁠. Analysis of the glycosylation and subclasses revealed that this is caused by lower fucosylation (afucosylation) and higher galactosylation of anti-S IgG and potentially a larger frequency of the IgG3 subclass [27,39,44]⁠. It was also shown that these afucosylated antibodies, together with S protein immunocomplexes, can elicit the release of pro-inflammatory cytokines and activate endothelium cells and platelets [45,46]⁠. In one study, deceased patients had lower levels of anti-S antibodies, and the antibody response was delayed in comparison to surviving ICU-admitted patients [28]⁠. However, the deceased patient cohort in this study was substantially older (37.1% older than 81 years in deceased patients vs. 7.9% in survivors). The IgG level decreases in individuals older than 71 years [47]⁠, and it cannot be excluded if the difference was simply a reflection of the age or had a causative role in the detrimental course of the deceased patients. The strong correlation of the IgG titer and afucosylated anti-S IgG titer with severe disease raises the question of whether IgG may be even more detrimental to the course of the disease. This hypothesis was raised in prior publications [13]⁠. While there are solid evidence-based associative data and in vitro studies to support this hypothesis, only two in vivo human and mouse studies show the potential detrimental roles of anti-SARS-CoV-2 antibodies. One clinical trial with the monoclonal antibody bavanalimab derived from recovered COVID-19 patients caused more severe symptoms in patients with a high viral load. Furthermore, in another trial, the coadministration of Fc-function defective estevimab prevented this negative effect [48]⁠. In another study on monoclonal antibodies in mice, it was shown that being dependent on anti-S IgG monoclonal antibodies can lead, at higher doses, to a worsening of weight loss after SARS-CoV-2 infection [49]⁠. Thus, few in vivo studies suggest that a potential detrimental effect of anti-SARS-CoV-2 antibodies exists under specific conditions and is dependent on the Fc domain. In most studies investigating Fc-mediated functions, the state of the effector cells in COVID-19 was not taken into consideration. Across many studies of severe COVID-19, there is dysregulation of the immune system with lymphopenia of T-cells and NK cells [50]⁠. Furthermore, these cell types show signs of exhaustion [51]⁠. On the other hand, there is an upregulation of neutrophils and a dysregulation of monocyte subsets. The FcγIIIa and FcγIIIb (CD16) receptor expression is upregulated in macrophages and neutrophils, and a FcγIIIa-expressing monocyte subtype was enriched in the airways of severely ill patients [52,53]⁠. Proteins of the complement system are also upregulated in severe COVID-19 patients [54]⁠. In most patients, there will be no more viremia when the IgG response is activated. However, multiple studies have shown that viral fragments and antigens like the S protein can persist after viremia [22,23,55]⁠. Thus, it could be that afucosylated anti-S IgG, together with the S protein as an antigen, in severe COVID-19 form immune complexes and will activate dysregulated monocytes, macrophages, the complement system, and neutrophils, thus contributing to the pathology by increasing the cytokine storm, hyperinflammation, and activation of thrombosis [Fig. 2].

## Factors determining antibody function in vivo based on studies administrating anti-SARS-CoV-2 antibodies

One important issue is under which conditions neutralizing or Fc-mediated functions are mediated in vivo. Innate immunity and T-cell adaptive immunity are both affected by natural infection and vaccination. One approach is to discern the antibody-mediated effects by investigating the effects of direct administration of mono- or polyclonal antibodies against SARS-CoV-2, either as treatment or prevention, in animal models. Detailed studies on the dependency of Fc-effector functions on monoclonal antibodies are mostly conducted in transgenic H18 ACE2 mice, which express the human ACE2 receptor, are susceptible to SARS-CoV-2, and can display a severe or fatal disease course. It should also be noted that there are major differences in the Fc receptor in mice compared to humans [14]⁠. However, there are also transgenic mice with humanized Fc-receptors [56]⁠.

### Dose dependency on neutralizing or Fc-mediated functions

**Fig. 3:**
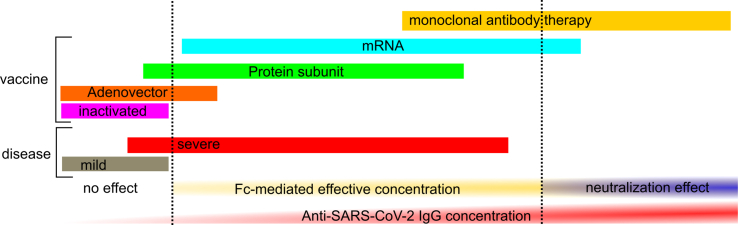
The range of antibody titers for Fc-dependent functions and neutralization for different vaccines and mild and severe disease, based on the effective range of monoclonal antibodies against SARS-CoV-2.

Several studies have investigated the dependence of the Fc-effector function by engineering monoclonal antibodies with an Fc-Null variant incapable of activating Fc receptors and comparing them with monoclonal antibodies with a functional Fc-domain. By doing so, the minimum therapeutic or prevention dose needed for protection from disease can be determined based on Fc-mediated effects or neutralization. Antibodies with functional Fc-domains needed minimum doses of 0.5–5 mg/kg, dependent on the monoclonal antibody and the Fc-domain structure, to reduce viral load, mortality rate, or weight loss [[56], [57], [58]]⁠. Furthermore, a non-neutralizing antibody with a fully functional Fc domain was effective at a dose of approximately 4 mg/kg [49]⁠. Fc-null anti-S antibodies were only effective at doses greater than 20–40 mg/kg [56,57,59]⁠. This indicates that Fc-mediated functions in anti-S IgG are effective at about 1/10 of the dose needed for neutralization-mediated effects. This makes it questionable whether neutralization is a biologically relevant effect outside of high-dose monoclonal antibody therapies, since 20 mg/kg monoclonal concentrations would lead to a serum antibody concentration greater than 100 μg/ml, which is only reached shortly after mRNA vaccination [60,61]⁠. However, the antibody kinetics are different between mice and humans, with a faster decline of antibodies in mice; therefore, the limit for neutralizing function may be different in humans [62,63]⁠. On the other hand, the minimum antibody dose per weight needed for effective antibodies is comparable in humans. From clinical trials, the lowest dose reported to be effective in reducing hospitalization rates was a single 300–700 mg dose, which would be a dose of 4.2–7 mg/kg for a 70 kg person [[64], [65], [66]]⁠. Studies on monoclonal antibodies in cancer therapy, which are dependent on the Fc-mediated antibody function, found a lower limit of 3 mg/kg for effective antibody effects [67]⁠. To compare these data to the antibody response of recovered or vaccinated individuals, the plasma or serum anti-S concentration must be compared with that of monoclonal antibody-treated individuals. Most studies on anti-SARS-CoV-2 IgG measure either the neutralization titer or the relative antibody titer to a reference value that is not standardized among different studies. Only a few studies on antibody titers in infection and vaccination have measured absolute anti-SARS-CoV-2 IgG concentrations. Furthermore, the plasma concentration of anti-S monoclonal antibodies has not been measured in most clinical trials. Estimation of bioavailability of other monoclonal antibodies involves estimating plasma antibody concentrations of 10–50 μg/ml 10 days after administration of a monoclonal antibody dose of 2–6 mg/kg body weight [62]⁠. While more research is needed to determine the range of effective anti-SARS-CoV-2 antibodies or viral antibodies in vivo, this estimation would set the lower range of effective anti-SARS-CoV-2 antibody concentration for a significant non-neutralization effect in protection higher than 10 μg/ml. In studies on COVID-19-recovered individuals, no individuals reached an anti-RBD IgG concentration higher than 10 μg/ml [68]⁠. In hospitalized patients, only a fraction had higher antibody levels past acute disease [61]⁠. Thus, in mild and moderate infections, the antibody response may not have a significant role either in disease resolution or protection, which is conferred by other mechanisms, such as the T-cell response [Fig. 3]. This is also supported by the lack of efficacy in the therapy of convalescent plasma from recovered patients [69]⁠. It may be possible that during the course of severe disease, significant anti-SARS-CoV-2 concentrations are reached, similar to mRNA vaccination [70]⁠ up to 100–5000 μg/ml [60]⁠, thus have the potential eliciting Fc-mediated functions. It is important to note that in in vitro neutralization assays, the maximum inhibition of infection is reached at 1 ug/ml [49,57]⁠, indicating that in vitro measurements are too sensitive and do not reflect the necessary concentration needed for in vivo conditions. Depending on the antibody, higher concentrations may not always increase function or provide greater protection. It is important to note that this effect may not be observed in all conditions. In one study in mice, there was a drop in binding to monocytes above a concentration of 10 μg/ml, and one neutralizing monoclonal antibody led to a worse disease outcome at higher doses, while lower doses were protective [49]⁠.

## IgG response by vaccines

### Vaccine types

During the COVID-19 pandemic, multiple vaccines for multiple vaccine platforms were rapidly developed for the first time against a coronavirus in humans. Most vaccines were based on the S glycoprotein as the sole antigen, with the exception of the inactivated virus, which uses the whole virus. The S glycoprotein of COVID-19 strains is either derived directly from the virus or can contain modified prolin substitutions (K986P and V987P) for stabilization, as well as mutations of the furin cleavage site to prevent the release of the S1 subunit [71]⁠. All vaccines are used in multiple-dose schemes. Usually, two doses spaced 28 days apart, and additional doses 3–6 months after the initial doses. All approved vaccine platforms are based on intramuscular injections [71,72]⁠.

### Inactivated vaccines

These vaccines are based on inactivated viral particles propagated in cell culture. To induce an immune response, they are co-administrated with adjuvants. The largest vaccine producers are in India (Batharat) and China (Sinopharm, Coronavac) [72]⁠.

### Adenovector vaccines

Adenovector vaccines use a replication-deficient adenovirus to transduce the target gene. These vaccines either utilize human adenovirus Ad26 and/or Ad5 (Sputnik V, Ad26Cov2-S, Ad5-nCoV) or a chimpanzee adenovirus strain (AZD1222). The vaccines encode either the Wuhan variant of the S protein (AZD1222, Sputnik V, Ad5-nCoV) or the stabilized version (Ad26Cov2-S) [72]⁠.

### Protein subunit vaccines

These are based on the direct application of the protein antigen, together with adjuvants, as immune stimulants. These could be the full-length S protein without the furin cleavage site and prolin stimulation or the S1 or RBD subunits. The most globally distributed vaccine is Novavax, but there are other locally developed vaccines [72,73]⁠.

### mRNA vaccines

The mRNA technology is based on the transfection of cells with modified RNA in lipid nanoparticles to express the desired protein—in this case, the S protein. The major producers are Biontech (BNT162b2) and Moderna (M1273). Both vaccines encode the stabilized version of the S glycoprotein. m1273 uses a higher dose of 100 μg vs. 30 μg in BNT162b2 [72]⁠.

### Early response (seroconversion-3 months after the first dose)

**Fig. 4:**
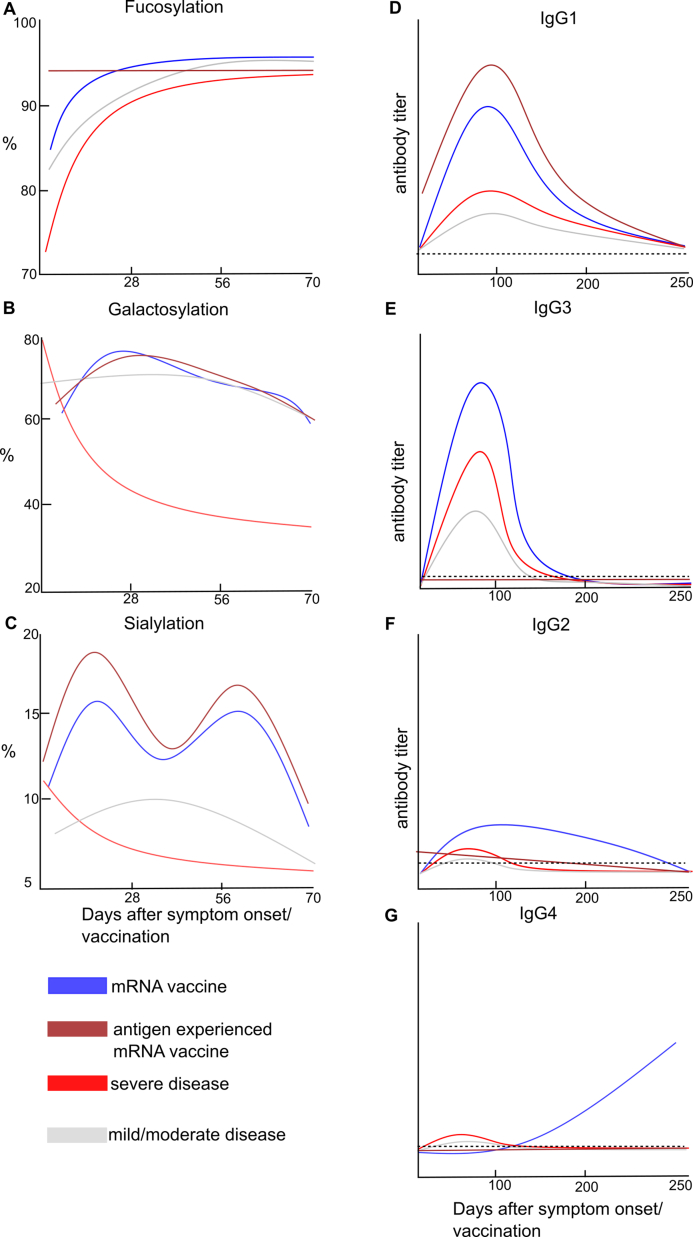
The trends of Glycosylation (**A-C**) and IgG subclass (**D-G**) of anti-spike IgG in mRNA COVID-19 vaccinated and infected individuals, adapted from Refs. [42,77,78]⁠. Antigen experienced refers to individuals who were already recovered from COVID-19 or received an non-mRNA COVID-19 vaccine dose before receiving mRNA COVID-19 vaccination. Dashed line in (**D-G**) represent the baseline as pre-pandemic spike protein binding IgG subclass.

The early antibody response after the first dose differs in magnitude and IgG profile, depending on the vaccine type. The highest anti-S IgG levels are reached by mRNA vaccine platforms, with similar or lower levels in protein subunit vaccines, and the lowest levels in adenovector and inactivated vaccines [5,60,74]⁠. If 10 μg/ml is set as the lower estimated limit (see section: Dose dependency on neutralizing or Fc-mediated functions) for any significant effect of anti-SARS-CoV-2 antibodies, then only mRNA and protein subunit vaccines should be able to elicit an antibody response with a protective effect [Fig. 3] [5,60,61]⁠. The antibody levels decline after the second dose and may fall below effective levels after 3 months [61]⁠. With respect to adenovector vaccines, only a fraction of individuals receiving the AstraZeneca vaccine (AZD1222, ChadOx1) reached anti-S antibody levels above 10 μg/ml [60]⁠, while the Johnson & Johnson vaccine (Ad26Cov2-S) had only a concentration of 1 μg/ml, similar to mild and moderate infection [61]⁠. Inactivated vaccines typically elicit antibody responses that are comparable or sometimes weaker in comparison to those observed in individuals who have recovered from COVID-19 [75,76]⁠. Inactivated and adenovector-based vaccines, which have the lowest efficacy among all vaccines, are likely to rely mostly on other mechanisms, like T-cell-mediated immunity, for protection. However, more research is needed to determine the effective range of anti-SARS-CoV-2 antibodies in vaccination. In mRNA vaccination, similar to natural infection, immune-naive individuals have a transient afucosylation of IgG1 [Fig. 4] [77,78]⁠. However, this is shorter (14 days until normalization in BNT vaccinated versus 42 days in ICU-admitted COVID-19 patients) and to a lesser degree (12% afucosylation at seroconversion in mRNA vaccinated vs. 20% in ICU-admitted patients). Furthermore, the data indicate that the antibody level is too low during the period of afucosylation to activate inflammation in macrophages via spike protein immune complexes [78]. The duration of lymphopenia observed in severe COVID-19 disease is shorter when individuals receive mRNA COVID-19 vaccination, and lymphocyte counts return to normal before seroconversion occurs [79]⁠. Additionally, other major changes in other leukocyte populations do not occur with mRNA vaccination. Only individuals who develop myocarditis after vaccination have aberrant leukocyte populations, but these are in many aspects the opposite in comparison to severe infection, with increased numbers of T-cells and NK cells and a decrease in FcγIIIa-expressing monocytes [80]⁠. Anti-S IgG in mRNA vaccination has a higher level of sialylation, which could lead to an increased expression of the inhibitory FcγIIb receptor and a subsequent decrease in inflammatory immune response [77,81]⁠. Thus, the IgG-mediated effector functions elicited by mRNA vaccination should be less inflammatory than in severe COVID-19. However, similar to severe COVID-19, there is a lack of research investigating the effects of the antibodies on the actual immune effector cells in infected or vaccinated individuals. If an individual was already recovered before vaccination or received an adenovector vaccination, no afucosylation occurred [77,78]⁠, indicating that afucosylation appears only in immune-naive individuals shortly after seroconversion. In adenovector and protein subunit vaccines, no afucosylation occurred after the first dose [77,81,82]⁠. While adenovector and inactivated vaccines themselves may not be sufficient for an effective immune response, they could still be useful in preventing IgG afucosylation as a first dose before the application of mRNA vaccines if afucosylation is not desired. One hypothesis based on the difference in fucosylation between enveloped viruses claimed that afucosylation is triggered by membrane-bound antigens and not by soluble protein antigens [13]⁠. However, the absence of afucosylation in adenovector vaccination, despite this vaccine should also induce membrane-bound antigens, contradicts this hypothesis. This suggests that other factors may also influence afucosylation. From animal-based studies, it is known that different adjuvants can alter the expression of glycosyltransferase as well as antibody glycosylation [19]⁠. mRNA vaccination itself can alter the innate immune system and the response to other diseases [83,84]⁠. However, it is unknown how the different vaccine platforms influence the glycosylation pathways. The IgG subclass distribution during the early antibody response in all vaccine platforms is similar to that of natural infection, with an IgG1-and IgG3-dominated response [Fig. 4] [77,85,86]⁠. One study suggests that there is a subgroup of mRNA-vaccinated individuals that have an IgG2-dominated response [87]⁠. IgG2 has less affinity for Fc receptors and should lead to weaker Fc-mediated functions [Fig. 1]. Thus, these individuals may have weaker protection from Fc-mediated functions.

### Long-term changes in antibody responses in vaccinated individuals

In the late antibody response to mRNA vaccines, there is a decline in anti-S IgG3 and a shift to the IgG4 subclass [Fig. 4] [60,77,88]⁠. However, the rise of IgG4 is impeded if the individual was infected with SARS-CoV-2 prior to mRNA vaccination or received an adenovector vaccination [77,88]⁠. Antigenic-specific IgG4 is usually elevated after repeated antigen exposure for immune tolerance to potential harmless antigens and therefore elicits only weak Fc-mediated responses [15]⁠. In accordance with this, it was found that high anti-S IgG4 sera from mRNA-vaccinated individuals have a weakened ADCP and complement activation [60]⁠. This implies that, with a rising IgG4 titer, higher anti-S IgG levels are needed for a significant Fc-mediated effect and may raise the minimum level for vaccine protection for later time periods. However, the exact role of elevated IgG4 on the antibody-mediated protection provided by mRNA vaccines is not yet clear. Elevated IgG4 titers are observed in latent and chronic viral infections like HIV or herpes virus infections and could be an expression of continuous antigen expression of viral proteins to which a shift of IgG4 happens over time [89,90]⁠. Indeed, there is evidence that expression of the S protein in mRNA vaccination can last more than 57 days after vaccination [91]⁠. In protein subunit vaccination against hepatitis B and parasitic or bacterial pathogens, the rise in antigen-specific IgG4 is well described [[92], [93], [94]]⁠⁠. High titers of IgG4 may even be protective against infection; for example, in a recent study on monkeys using Novavax as a third dose after two doses of mRNA vaccination, a predominant IgG4 response was observed, leading to lower viral titers and inflammation after viral challenge [95]⁠. On the other hand, elevated total IgG4 or anti-S IgG4 levels were associated with higher mortality rates in unvaccinated individuals [96,97]⁠. More research is needed to investigate the overall impact of the subclass shift in IgG4. Furthermore, there is a need to study the regulatory pathways involved in the IgG subclass shift within new vaccination platforms.

## Influence of SARS-CoV-2 variants on Fc-mediated functions

Similar to other coronavirus strains, SARS-CoV-2 mutates, and the antigen structure shifts over time, especially if selection pressure is present to alter the antigen for immune evasion [12]⁠. The COVID-19 vaccines were all directed against the original Wuhan strain and provided protection against infection and disease from different variants. The protection slightly declined with the appearance of the delta variant, and most protection was lost with the omicron variant, which had more than 30 point mutations in the S protein. In the case of variants of concern, most studies have shown remarkable stability of ADCP, complement activation, and ADCC to different variants [[98], [99], [100]]. In pre-omicron variants, both mRNA-vaccinated individuals and those who had recovered from COVID-19 demonstrated robust activity in in vitro assays for all Fc-mediated functions against multiple variants. This activity was found to be comparable to the activity observed against the beta and delta variants. Only in the Brazilian P1 variant was there a significant drop in Fc-mediated functions [87]⁠. For the omicron variants, a study on individuals who received mRNA vaccines, Coronavac, or had previously recovered from COVID-19, found a decrease in ADCC activity and Fc-receptor affinity in anti-RBD antibodies, while the full S protein antibodies did not exhibit such changes [99]⁠. Another study found a drop in ADCP and ADNP from the Wuhan variant to different omicron variants in inactivated vaccines [75]⁠.

## Conclusions

In summary, the current evidence shows that the effects of anti-SARS-CoV-2 antibodies in disease protection and the potential pathogenic effects of IgG in severe disease and vaccination are primarily driven by Fc-mediated functions rather than neutralizing effects. It is important to note that the concentrations of anti-SARS-CoV-2 antibodies may only reach levels that have significant effects in mRNA and protein subunit vaccination, monoclonal antibody therapies, and severe disease, rather than inactivated and adenovector vaccines or mild to moderate symptoms. However, the lack of standardized methods for measuring antibody functions, as well as the challenges in translating these findings into in vivo conditions, restricts the use of the current data to predict the effectiveness of specific methods for disease protection. Furthermore, there is a lack of research on how overall antibody-mediated functions evolve during different stages of the disease and the status of antibody-effector cells.

## Conflicts of interest

The authors declare no conflict of interests.
